# Predicting Prognostic Effects of Acupuncture for Depression Using the Electroencephalogram

**DOI:** 10.1155/2022/1381683

**Published:** 2022-03-02

**Authors:** Xiaomao Fan, Xingxian Huang, Yang Zhao, Lin Wang, Haibo Yu, Gansen Zhao

**Affiliations:** ^1^School of Computer Science, South China Normal University, Guangzhou, China; ^2^Department of Acupuncture and Moxibustion, Shenzhen Traditional Chinese Medicine Hospital, Shenzhen, China; ^3^School of Data Science, City University of Hong Kong, Hong Kong SAR, China; ^4^Shenzhen Institutes of Advanced Technology, Chinese Academy of Sciences, Beijing, China

## Abstract

Depression is considered to be a major public health problem with significant implications for individuals and society. Patients with depression can be with complementary therapies such as acupuncture. Predicting the prognostic effects of acupuncture has a big significance in helping physicians make early interventions for patients with depression and avoid malignant events. In this work, a novel framework of predicting prognostic effects of acupuncture for depression based on electroencephalogram (EEG) recordings is presented. Specifically, EEG, as a widely used measurement to evaluate the therapeutic effects of acupuncture, is utilized for predicting prognostic effects of acupuncture. Max-relevance and min-redundancy (mRMR), with merits of removing redundant information among selected features and remaining high relevance between selected features and response variable, is employed to select important lead-rhythm features extracted from EEG recordings. Then, according to the subject Hamilton Depression Rating Scale (HAMD) scores before and after acupuncture for eight weeks, the reduction rate of HAMD score is calculated as a measure of the prognostic effects of acupuncture. Finally, five widely used machine learning methods are utilized for building the predicting models of prognostic effects of acupuncture for depression. Experimental results show that nonlinear machine learning methods have better performance than linear ones on predicting prognostic effects of acupuncture using EEG recordings. Especially, the support vector machine with Gaussian kernel (SVM-RBF) can achieve the best and most stable performance using the mRMR with both evaluating criteria of FCD and FCQ for feature selection. Both mRMR-FCD and mRMR-FCQ obtain the same best performance, where the accuracy and *F*_1_ score are 84.61% and 86.67%, respectively. Moreover, lead-rhythm features selected by mRMR-FCD and mRMR-FCQ are analyzed. The top seven selected lead-rhythm features have much higher mRMR evaluating scores, which guarantee the good predicting performance for machine learning methods to some degree. The presented framework in this work is effective in predicting the prognostic effects of acupuncture for depression. It can be integrated into an intelligent medical system and provide information on the prognostic effects of acupuncture for physicians. Informed prognostic effects of acupuncture for depression in advance and taking interventions can greatly reduce the risk of malignant events for patients with mental disorders.

## 1. Introduction

Depression is one of the most common mental disorders, characterized by a persistent low mood, loss of interest, or reduced energy. According to the World Health Organization's (WHO) report, over 264 million persons at all ages suffer from depression [1, 2]. Depression has a serious impact on the person's study, work, and social life. In the worst case, depression can cause the affected person to self-harm or even commit suicide when she/he is under long-lasting moderate or severe intensity [3]. Acupuncture has good clinical efficacy in the treatment of depression, with no side effects or adverse reactions. However, the mechanism of acupuncture treatment is still controversial and there is still no objective evidence for evaluating its therapeutic effects [4, 5]. The Hamilton Depression Rating Scale (HAMD) [6] is a widely used tool to assess the efficacy of depression treatment. Its assessment is quite subjective and requires a trained physician; therefore, designing an objective method to assess the efficacy of depression treatment has great significance.

Electroencephalogram (EEG) is a common tool to capture the electrical activity changes caused by acupuncture [7]. Specifically, in the central nervous system of the human brain, there are many neurons. Moreover, those neurons will continuously produce potential changes. Through EEG equipment, EEG signals can be acquired by recording the electrical activity of the aforementioned neurons from the scalp [8]. Many researchers attempted to utilize EEG recordings to identify depression [9–12]. Cai et al. [10] presented a case-based reasoning model for identifying depression with three-electrode EEG signals. Steiger et al. [12] analyzed EEG characteristics from an individual during the wake and sleep phase and provided a series of biomarkers to screen depression. Hosseinifard et al. [11] investigated a nonlinear analysis method for EEG signals to identify depression patients and normal individuals. Acharya et al. [9] employed a state-of-the-art convolutional neural network (CNN) to screen patients with depression based on EEG signals. Meanwhile, researchers also utilized EEG signals to evaluate the therapeutic effects of depression [13, 14]. Noemi et al. [13] investigated the brain oscillations in the gamma frequency band of EEG signals and evaluated the therapeutic effects with selective serotonin reuptake inhibitors (SSRIs) for depression. Shadli et al. [14] studied the effects of ketamine on the EEG of patients with treatment-resistant generalized anxiety and social anxiety disorders. Since EEG has different signal morphological patterns on patients with depression or not [15, 16], it is shown from experiment and analysis results that all of the aforementioned studies achieved a good performance using EEG recordings on screening patients with depression and evaluating the therapeutic effectiveness of antidepressants. Therefore, EEG can also be utilized to evaluate the prognostic effects of acupuncture, which enables physicians to understand the prognosis and health conditions of patients in advance.

In this article, we proposed a framework based on EEG recordings with machine learning methods for predicting the prognostic effectiveness of acupuncture for depression. Specifically, lead-rhythm features extracted from EEG recordings are selected by max-relevance and min-redundancy (mRMR) with both evaluating criteria of FCD and FCQ. Then, according to the subjects' HAMD scores before and after acupuncture for eight weeks, the reduction rate of HAMD scores is calculated as a measure of the prognostic effects of acupuncture. Finally, popular machine learning methods of logistic regression (LR), random forest (RF), and support vector machine with the linear kernel (SVM-Linear), poly kernel (SVM-Poly), and Gaussian kernel (SVM-RBF) are employed to build 8-week predicting models of prognostic effectiveness of acupuncture for depression. With the help of the predicting model of prognostic effects of acupuncture, physicians can learn about their patients' mental conditions in advance and take necessary interventions to reduce the risk of malignant events.

## 2. Materials and Methods

**Figure 1:**
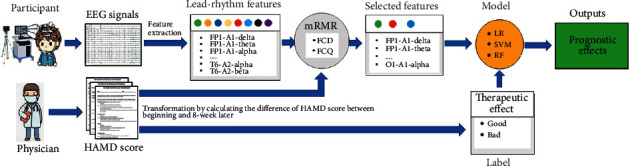
Overview of the predicting framework of prognostic effects of acupuncture for depression with EEG recordings.

Figure 1 shows the proposed predicting framework of prognostic effects of acupuncture for depression. It mainly consists of EEG recording acquisition, feature selection with the mRMR [17, 18], and predicting models built by widely used machine learning methods. The details are described as follows.

### 2.1. Participants

Subjects with depression who were admitted to Shenzhen Traditional Chinese Medicine Hospital for inclusion criteria were collected. This study was approved by the Ethics Committee of the Shenzhen Traditional Chinese Medicine Hospital with the IRB Number of 2017-8. All the patients signed informed consent and voluntarily participated in this clinical study. Subjects inclusion criteria include the following: (1) meeting the western medical diagnostic criteria for digression; (2) conscious; (3) over 18 years old or under 65 years old. Meanwhile, subjects are excluded under the following conditions: (1) schizophrenia, organic diseases, and physical diseases; (2) pregnant women; (3) those with severe liver and kidney function, cardiovascular and cerebrovascular diseases, and hematopoietic system diseases; (4) not cooperating with acupuncture treatment and poor drug compliance. Regarding treatment intervention, physicians employ acupuncture for depression treatment with the technique of transferring and regulating acupuncture [19], which is proposed by our research team: five times a week, a four-week course of treatment, and a total of 2 courses of acupuncture treatment.

#### 2.1.1. Depression Assessment

The 17-item HAMD [6] is the most widely used scale for depression assessment at present. It has good reliability and validity and can reflect the changes of depression symptoms in a relatively sensitive manner. It is one of the best assessment tools in therapeutic research and can better reflect the severity of depression. Therefore, we utilize the HAMD-17 score to assess the prognostic effects of acupuncture for depression.

### 2.2. EEG Recording Acquisition

**Figure 2:**
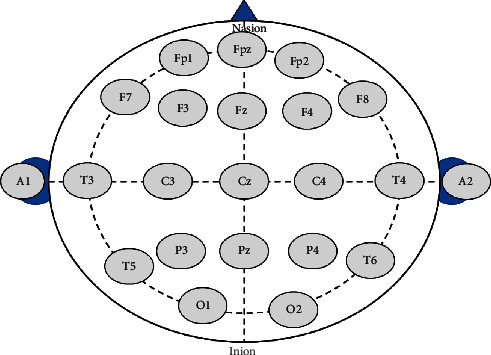
International “10-20” system, which describes the location of scalp electrodes of EEG signals.

A Neuron-Spectrum-5 EEG device manufactured by Neursoft Ltd., Russia, is used to collect EEG recordings from patients with depression syndrome. The room temperature is controlled at 18–25°C. Scalp electrodes are placed in accordance with the international 10/20 system electrode placement method (Figure 2), and bilateral ear lobes are used as the reference electrodes to record 19 conductive EEG signals. EEG was collected under the condition of quiet eye closure and relaxation. Steady EEG signals are collected before acupuncture (https://www.medicalexpo.com/prod/neurosoft/product-69506-454186.html).

### 2.3. Features

#### 2.3.1. Feature Description

Figure 2 shows the distribution of scalp electrodes of EEG signals. In this study, EEG recordings we collected consist of 19-lead signals, which are presented as FP1-A1, FP2-A2, F3-A1, F4-A2, FZ-A2, C3-A1, C4-A2, CZ-A1, P3-A1, P4-A2, PZ-A2, O1-A1, O2-A2, F7-A1, F8-A2, T3-A1, T4-A2, T5-A1, and T6-A2. FP1-A1, for example, means that the lead signal is acquired from scalp electrodes of FP1 and A1. Four rhythm features (signal amplitude) of delta (*δ*), theta (*θ*), alpha (*α*), and beta (*β*) are extracted by the embedded digital EEG system in Neuron-Spectrum-5 EEG device. In this case, there are a total of 76 lead-rhythm features for subsequent processing. The lead-rhythm features can be presented in the form of “(lead)-(rhythm).” For example, the four rhythm indexes of lead FP1-A1 can be described as FP1-A1-delta, FP1-A1-theta, FP1-A1-alpha, and FP1-A1-beta, respectively.

#### 2.3.2. Prognostic Effect Evaluation

In this study, the 17-item HAMD rating scale (HRS) is utilized to assess depression conditions for each subject before acupuncture and after acupuncture for eight weeks. To assess the prognostic effects of acupuncture for depression, the reduction rate *R*_*HRS*_ of HAMD scores with the difference of HAMD scores of two times on each subject is used to evaluate the prognostic therapeutic effects [20], which is defined as follows:

(1) $$\begin{array}{c} R_{HRS}=\frac{D{\text{Score}}_{\text{pre}}-D{\text{Score}}_{\text{pos}}}{D{\text{Score}}_{\text{pre}}}, \end{array}$$

where *D*Score_pre_ refers to the HAMD score before acupuncture and *D*Score_pos_ refers to the HAMD score of acupuncture for eight weeks. Here, to simplify the problem of predicting prognostic effects, the reduction rate of HAMD scores is mapped into binary values 0 and 1. If *R*_*HRS*_ is greater than 0.5, the class label *y* of the prognostic effect is defined as 1, which means good prognostic effects. Otherwise, *y* is defined as 0, which means bad prognostic effects.

#### 2.3.3. Feature Selection and Normalization

Feature selection is a very important procedure before building a predicting model of prognostic effects of acupuncture for depression when the sample size is not more than the number of features. The mRMR [17], as one of the popular feature selection methods, is with the advantages of removing redundancy information among selected features and keeping high relevance between selected features and response variables. The relevance and redundancy information of mRMR can be measured by mutual information for category data features and class labels. For continuous data features and category labels, the relevant information is measured by F-statistic and Pearson's correlation coefficients. In this study, the lead-rhythm features are continuous variables and class labels of prognostic effects are binary category variables. Therefore, the mRMR in this study employs F-statistic and Pearson's coefficients to measure the relevance and redundancy information among selected lead-rhythm features and class labels.

Generally, given two random vectors *x* and *y* with continuous values, the Pearson correlation coefficients *P*(*f*_*i*_, *f*_*j*_) can be defined as follows:

(2) $$\begin{array}{c} P\left(f_{i},f_{j}\right)=\frac{E\left(f_{i}f_{j}\right)-E\left(f_{i}\right)E\left(f_{j}\right)}{\sqrt{E\left(f_{i}^{2}-E^{2}\left(f_{i}\right)\right)}\sqrt{E\left(f_{j}^{2}\right)-E^{2}\left(f_{j}\right)}}, \end{array}$$

where *f*_*i*_ and *f*_*j*_ are two features in the selected lead-rhythm feature set. Therefore, minimum-redundancy information (*M* Red) of selected lead-rhythm features *S* can be defined as follows:

(3) $$\begin{array}{c} M\text{ Red}\left(S\right)=\min\frac{1}{{\left\|S\right\|}^{2}}\sum_{f_{i},f_{j}\in S}\left|P\left(f_{i},f_{j}\right)\right|. \end{array}$$

To obtain maximum-relevance information *M* Rel between selected features *S* and class label vector *y*, it can be defined as follows:

(4) $$\begin{array}{c} M\text{ Rel}\left(S,y\right)=\max\frac{1}{\left\|S\right\|}\sum_{f_{i}\in S}F\left(f_{i},y\right), \end{array}$$

where *F* is the F-statics, which can be defined as follows:

(5) $$\begin{array}{c} F\left(f_{i},y\right)=\frac{\left(n-K\right)\sum_{k}n_{k}{\left(\bar{m_{ik}}-\bar{m_{i}}\right)}^{2}}{\left(K-1\right)\sum_{k}\sigma_{k}^{2}}, \end{array}$$

where *m*_*ik*_ is the mean value of the *i*-th selected feature within the *k*-th class (*k*=1,…*K*); *m*_*i*_ is the mean value across all entries in the *i*-th selected feature; *σ*_*k*_ is the variance of the *k*-th selected features across all data entries.; *n* is the size of the whole data entries and *n*_*k*_ is the size of the data entries in the *k*-th class; *K* is the number of class labels. There are two ways to obtain maximum-relevance minimum-redundancy information, namely, mRMR evaluating score, with *F*-test correlation difference (FCD) and the *F*-test correlation quotient (*FCQ*), which can be defined as follows, respectively:

(6) $$\begin{array}{c} FC\;D=M\text{ Rel}\left(S,y\right)-M\text{ Red}\left(S\right), \end{array}$$

(7) $$\begin{array}{c} FCQ=M\text{ Rel}\left(S,y\right)\backslash M\text{ Red}\left(S\right). \end{array}$$

Due to a large variation in terms of amplitudes among lead-rhythm selected features, it is necessary to utilize a normalization technique to map the selected features into a uniform range. In this study, the Min–Max normalization technique with the range from 0 to 1 is employed, which is defined as follows:

(8) $$\begin{array}{c} \text{Norm}\left(f_{i}\right)=\frac{f_{i}-f_{\min}}{f_{\max}-f_{\min}}, \end{array}$$

where *f*_*i*_ is the *i*-th selected lead-rhythm feature, *f*_max_ refers to the maximum value of the *f*_*i*_, and *f*_min_ refers to the minimum value of the *f*_*i*_.

### 2.4. Machine Learning Methods

In this study, popular machine learning methods of logistic regression, random forest, and support vector machine are utilized to build predicting model of prognostic effects of acupuncture for depression based on EEG recordings. The details are described as follows.

#### 2.4.1. Logistic Regression

Logistic regression (LR) is a classification method that utilizes the Sigmoid function as a posterior probability distribution to classify the input data [21]. The LR can be used for both binary and multiple classification problems. On the other hand, the LR is easy to be implemented and can be applied to both distributed and real-time application scenarios. In this study, we utilize the default threshold value of 0.5 to classify the good and bad prognostic effects of acupuncture. Specifically, if the probability of the LR output is more than the threshold, the prognostic effect should be predicted to be good prognostic effect; otherwise, it is a bad prognostic effect.

#### 2.4.2. Random Forest

Random forest (RF) is an ensemble classifier that contains multiple decision trees [22]. It is widely used for variant classification tasks and achieves quite well performance. Specifically, given a training dataset *D* with sample size *N*, the RF employs the bootstrap resampling technique to repeatedly extract *k*(*k* < *N*) samples from the original training dataset. The extracted datasets are the new training datasets for decision trees. If the dimension of each sample is *M*, specify a constant *m*(*m* ≤ *M*) and randomly select *m* feature subsets from *M* features. In this study, we employ the model of CART as the base decision tree model [23], which uses the minimum criterion of the Gini index for optimal feature selection. Train the base decision trees based on the newly generated training data subsets, and the major voting method is utilized for all the base decision tree models to make the final prediction.

#### 2.4.3. Support Vector Machine

Support vector machine (SVM) is a binary classification model, which searches a hyperplane with the largest support vector margin in affine high-dimensional feature space with kernel techniques [24]. Given a training dataset *D*, the SVM aims to search a separate hyperplane in terms of the training dataset to classify samples into their class groups as many as possible. Furthermore, to map original data features to higher feature dimensions, a total of three kinds of different kernels of linear, poly, and Gaussian are utilized for predicting prognostic effects in this article, which are called SVM-Linear, SVM-Poly, and SVM-RBF in short, respectively.

### 2.5. Performance Metrics

In this article, four classification performance metrics of precision (Pre), recall (Rec), accuracy (Acc), and *F*1 score are employed to evaluate the predicting models of prognostic effects of acupuncture. As shown in Table 1, *TP* means the number of subjects with good prognostic effects predicted to be ones with good prognostic effects. *FP* means the number of subjects with bad prognostic effects predicted to be ones with good prognostic effects. *FN* means the number of subjects with good prognostic effects predicted to be ones with bad prognostic effects. *TN* means the number of subjects with bad prognostic effects predicted to be ones with bad prognostic effects. Therefore, the aforementioned performance metrics can be defined as follows:

(9) $$\begin{array}{c} \text{Pre}=\frac{TP}{TP+FP},\\ \text{Rec}=\frac{TP}{TP+FN},\\ \text{Acc}=\frac{TP+TN}{TP+TN+FP+FN},\\ F1=\frac{2\cdot \text{Pre}\cdot \text{Rec}}{\text{Pre}+\text{Rec}}. \end{array}$$

## 3. Results

### 3.1. Computing Environment

All experiments are conducted on a computing server equipped with a 20-core Intel Xeon E2660 v2 processor and 252 GByte memory. Regarding the three machine learning methods, they run on the machine learning platform of SKlearn V0.23.1, which is deployed in the CentOS 6.5 operation system.

### 3.2. Data Source

In this article, 92 subjects (age: 37.58 ± 10.99 years; gender: 31 male and 61 female) satisfied inclusion criteria are recruited from Shenzhen Traditional Chinese Medicine Hospital to collect EEG data and HAMD scores. A physician of Shenzhen Traditional Chinese Medicine Hospital takes charge of this data collection lasting almost one year from October 2018 to May 2019. Especially for HAMD scores collected, each subject has to be inquired with the HRS form two times. The first HAMD score is obtained before the acupuncture; the second one is obtained by a follow-up way after acupuncture for eight weeks. However, most of the recruited subjects are lost contacts or did not want to go to the hospital to complete the test and only 26 subjects with completed EEG recordings and two times HAMD scores remained. In other words, there are 26 observations we use to build the predicting model of prognostic effects of acupuncture in this article.

### 3.3. Classification Performance

In this section, leave-one-out cross-validation is utilized to evaluate the classification performance of the predicting models of the prognostic effect of acupuncture for depression. Specifically, only one observation is left at a time as the test dataset, and the remaining observations as the training dataset are used for the training predicting model of prognostic effect. Given that the size of the dataset is *N*, the predicting model of prognostic effect is needed to train *N* times and test *N* times. However, the classification performance of prognostic effects of acupuncture is calculated according to the accumulated test results from each validation test.

To train the predicting models of prognostic effects of acupuncture, five popular machine learning methods of LR, RF, SVM-Linear, SVM-Poly, and SVM-RBF are implemented with important hyperparameters listed in Table 2. Specifically, for the LR model, there are three hyperparameters of tolerance, solver, and iterated epochs. In order to obtain the best classification performance of the LR model, tolerance is set to be 1*e*^−4^ and the iterated epochs are to be 100. To overcome the small size problem, we utilize “newton-cg” as the solver of the LR model. For the RF model, hyperparameters of a number of estimators, split criterion, and a number of features to train are important for its classification performance. In this study, they are set to be 100, “gini,” and “sqrt,” respectively. Regarding the SVM model, three respective kernels are employed to train a predicting model of prognostic effects of acupuncture. These three kernels are the linear kernel, poly kernel, and Gaussian kernel. Excluding different kernels of the SVM-Linear, SVM-Poly, and SVM-RBF model, there are also some specified hyperparameters for each predicting model. For example, the SVM-Poly has the hyperparameter of the degree to be set, which determines the order of the poly kernel. Here, we set the degree to be 3. For the SVM-RBF, it has a hyperparameter of gamma to be set. The gamma is mainly used to map the height of low-dimensional samples. The higher the gamma is, the higher the mapped dimension is. In this study, the gamma is set to be “scale,” which is defined as follows:

(10) $$\begin{array}{c} \text{gamma}=\frac{1}{N_{\text{fea}}\sigma }, \end{array}$$

where *N*_fea_ is the number of features and *σ* is the variance of the training data.

**Figure 3:**
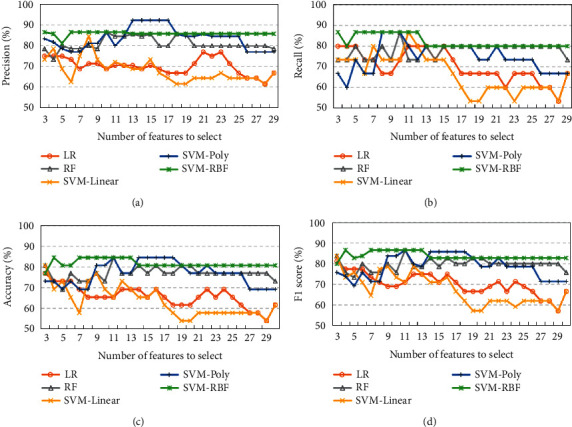
Classification performance of machine learning methods with the number of features to select using mRMR of FCD. (a) Precision. (b) Recall. (c) Accuracy. (d) F1 score.

**Figure 4:**
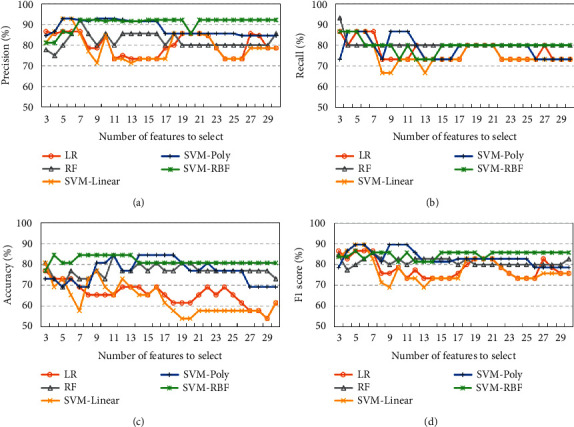
Classification performance of machine learning methods with the number of features to select using mRMR of FCQ. (a) Precision. (b) Recall. (c) Accuracy. (d) F1 score.

It is noted that the number of features to select is critical to the predicting models of prognostic effects of acupuncture. However, there are no criteria to determine the number of selected features in the mRMR itself. Therefore, the number of selected features is determined by a wrapper technique with the predicting models. First of all, concerning the sample size of the training dataset, the number of selected features of the mRMR ranges from 3 to 30 in this work. Then, the selected lead-rhythm features are ranked in descending order according to the scores of mRMR. Finally, prognostic effect models of acupuncture are built with the machine learning methods of LR, RF, SVM-Linear, SVM-Poly, and SVM-RBF. As shown in Figures 3 and 4, the classification performance of precision, recall, accuracy, and F1 score varies a lot with the number of features to select using the mRMR of FCD and FCQ. Obviously, nonlinear models of RF, SVM-Poly, and SVM-RBF are better than linear models of LR and SVM-Linear in predicting prognostic effects of acupuncture regardless of whether mRMR-FCD or mRMR-FCQ is used. Additionally, among those nonlinear models, the SVM-RBF can achieve better and more stable classification performance. Specifically, the SVM-RBF can obtain its best performance with mRMR-FCD and mRMR-FCQ. For the mRMR-FCD, precision is 86.66%, recall is 86.66%, accuracy is 84.61%, and F1 score is 86.67%. Regarding the mRMR-FCQ, precision is 92.30%, recall is 86.66%, accuracy is 84.61%, and F1 score is 86.67%. There is no obvious difference of classification performance of the SVM-RBF with mRMR-FCD and mRMR-FCQ.

## 4. Discussion

**Figure 5:**
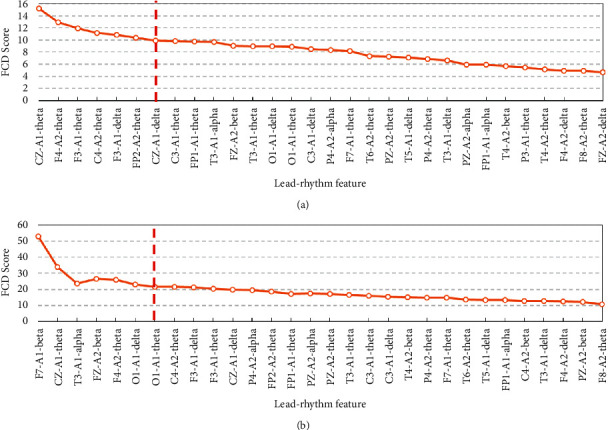
Score trend chart of lead-rhythm features to select. (a) FCD. (b) FCQ.

**Figure 6:**
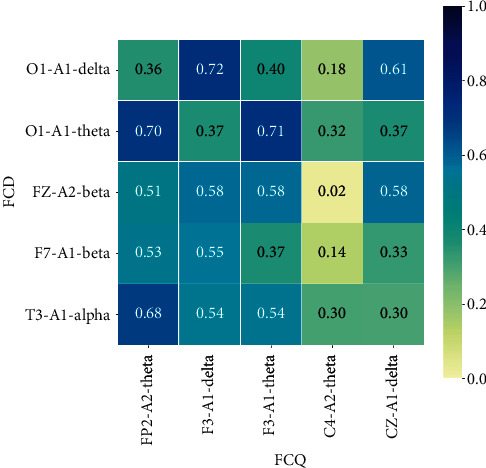
Heatmap of correlation coefficients between selected lead-rhythm features with FCD and FCQ.

Figures 3 and 4 show that the SVM-RBF can achieve quite well classification performance when the number of features to select is 7 in both mRMR-FCD and mRMR-FCQ. As shown in Figure 5, most of lead-rhythm features with high mRMR scores, which are calculated by equations (6) and (7), are in the top 7. In terms of the mRMR-FCD, the top 7 features to select are “CZ-A1-theta,” “F4-A2-theta,” “F3-A1-theta,” “C4-A2-theta,” “F3-A1-delta,” “FP2-A2-theta,” and “CZ-A1-delta.” For the mRMR-FCQ, the top seven features to select are “F7-A1-beta,” “CZ-A1-theta,” “T3-A1-alpha,” “FZ-A2-beta,” “F4-A2-theta,” “O1-A1-delta,” and “O1-A1-theta.” Among the top seven features to select, the lead-rhythm features of “CZ-A1-theta” and “F4-A2-theta” are selected by both mRMR-FCD and mRMR-FCQ. As for the remaining ten different selected features, mRMR-FCD and mRMR-FCQ have five respective features. In addition, to learn about the causes of why mRMR-FCD and mRMR-FCQ select different lead-rhythm features, we investigate the relationship among the selected features from mRMR-FCD and mRMR-FCQ. According to the theory of the mRMR, it minimizes the redundancy among selected features. In other words, a selected feature can be substituted by another one if they have a high correlation between them. Therefore, correlation coefficients of selected features between mRMR-FCD and mRMR-FCQ are calculated, which is shown in Figure 6. It is noted that the selected features from mRMR-FCD have at least one feature selected by mRMR-FCQ with a high correlation coefficient. For instance, the selected feature “O1-A1-delta” from mRMR-FCD has a high relationship with the selected feature “F3-A1-delta” from mRMR-FCQ; the correlation coefficient is 0.72. For the selected feature “F7-A1-beta” from mRMR-FCD, there are two selected features of “FP2-A2-theta” and “F3-A1-delta” from mRMR-FCQ with a high relationship. The correlation coefficients are 0.53 and 0.55, respectively. It means that the selected lead-rhythm features with a high relationship can be replaced with each other to some degree. Namely, there is no big difference between mRMR-FCD and mRMR-FCQ to select crucial lead-rhythm features even if they have respective mRMR score mechanisms.

Meanwhile, it is noted that most of the selected top lead-rhythm features using mRMR-FCD and mRMR-FCQ are with theta and delta rhythm, both of which belong to the slow wave. The theta rhythm is related to the amygdaloid nucleus, hippocampus, and thalamus from the limbic system [25]. The occurrence of theta wave is a manifestation of central nervous system depression, which is closely related to mental state, cognition and emotion. On the other hand, most of the selected important lead-rhythm features are from the leads of “F3,” “F4,” “FP2,” “F7,” and “FZ,” which are located at the frontal and central region of a human brain. The prefrontal cortex plays a key role in determining psychopathological susceptibility [26]. For example, selected lead-rhythm features of “F3-A1-theta” and “F4-A2-theta” are located on the dorsolateral prefrontal cortex, which are verified to be significant to the occurrence of depression [27, 28].

To sum up, the main contributions of this article are concluded as follows. (1) We present a novel framework of predicting prognostic effectiveness of acupuncture for depression with EEG recordings. (2) The mRMR with the advantages of removing redundant features and keeping maximum relevance with the response variable is utilized to select important lead-rhythm features. Meanwhile, the reduction rate of HAMD score as a measure of prognostic effects of acupuncture is employed to produce the class labels with a threshold value of 0.5. (3) Widely used machine learning methods of LR, RF, SVM-Linear, SVM-Poly, and SVM-RBF are employed to build the 8-week predicting model of prognostic effects of acupuncture for depression. (4) Experiment results show that the proposed framework with nonlinear machine methods of RF, SVM-Poly, and SVM-RBF outperforms that with linear machine learning methods of LR and SVM-Linear. Furthermore, selected lead-rhythm features from mRMR-FCD and mRMR-FCQ are analyzed for clinical functions related to depression.

## 5. Conclusions

In this article, a novel framework for predicting prognostic effects of acupuncture for depression with EEG recordings is presented. More specifically, the mRMR, with merits of minimum redundancy among lead-rhythm features and maximum relevance between lead-rhythm features and prognostic effects, is utilized to select important lead-rhythm features. Meanwhile, the reduction rate of HAMD score is calculated and binarized to the prognostic effect label. Widely used machine learning methods of LR, RF and SVM are employed to build the predicting models of prognostic effects of acupuncture. Extensive experiments show that the presented framework of prognostic effects of acupuncture for depression can achieve well performance, where the best accuracy and *F*1 score are 84.61% and 86.67%, respectively. Furthermore, the selected important lead-rhythm features from mRMR-FCD and mRMR-FCQ are analyzed with correlationship technique, and we find that there is a strong relationship between lead-rhythm features selected by mRMR-FCD and mRMR-FCQ. Therefore, the presented framework can help physicians and health providers learn about patients' mental conditions in advance and make essential interventions to reduce the risk of malignant events.

## Figures and Tables

**Table 1:** Confusion matrix of four metrics for classification performance.

Confusion matrix		Truth	
		Positive	Negative
Prediction	Positive	TP	FP
	Negative	FN	TN

**Table 2:** Hyperparameter configuration of machine learning methods.

Methods	Optimized hyperparameter settings
LR	Tolerance: 1*e*^−4^; solver:“Newton-cg”;
	Iterated epochs:100.
RF	No. of estimators:100; criterion = “gini”;
	Max features: “Sqrt”.
SVM-linear	kernel:“linear”; tolerance: 1*e*^−3^.
SVM-poly	kernel:“poly”; tolerance: 1*e*^−3^; degree: 3.
SVM-RBF	kernel:“rbf”; tolerance: 1*e*^−3^;
	Gamma: “Scale”.

## Data Availability

According to the requirement from the IRB of Shenzhen Traditional Chinese Medicine Hospital, all the data cannot be public due to the concerns of patients' privacy.
